# Bacteriophage Therapy: Overcoming Antimicrobial Resistance Through Advanced Delivery Methods

**DOI:** 10.3390/molecules31020324

**Published:** 2026-01-17

**Authors:** Marcin Wacnik, Emilia Hauza, Aneta Skaradzińska, Paulina Śliwka

**Affiliations:** Department of Biotechnology and Food Microbiology, Wrocław University of Environmental and Life Sciences, Chełmońskiego 37, 51-630 Wrocław, Poland

**Keywords:** bacteriophages, phage therapy, AMR, delivery strategies, routes of administration, liposomes, hydrogels

## Abstract

Microbial resistance to antibiotics necessitates the development of alternative treatments to address the challenges posed by severe bacterial infections. Bacteriophages are regaining clinical relevance, but the effectiveness of phage therapy depends directly on the route of administration and the carrier used. This review provides a critical overview of the therapeutic potential of phages, emphasizing different strategies for delivery to the site of infection. We focus on the preclinical and clinical data on phage therapies using various routes of administration, such as oral, intravenous, inhalation, topical, and local administration to joints and bones. In view of different phage formulations, including liquid suspension, phages immobilized in polymers or liposome-based carriers, we highlight the potential challenges and obstacles that may affect phage stability and bioavailability and limit the successful outcome of therapy. This review serves to enhance the understanding of the integration of materials engineering with clinical practice and production standardization, to address these issues. Additionally, a clear knowledge of the bacteriophage and pharmacokinetics of phage preparations is necessary to implement safe and efficacious bacteriophage treatment in the era of antimicrobial resistance.

## 1. Introduction

Antimicrobial resistance (AMR) remains a major global health concern and continues to challenge the effective treatment of bacterial infections. Recent estimates indicate that infections caused by antibiotic-resistant bacteria were responsible for over one million deaths annually and contributed to several million additional deaths worldwide [1,2]. Although projections suggest that this burden may further increase in the coming decades [3], the limitations of current antimicrobial strategies extend beyond resistance alone. The slow pace of antibiotic discovery, high development costs, and limited commercial incentives underscore the urgent need for complementary and alternative antibacterial approaches [1,4,5,6].

Bacteriophage therapy has re-emerged as a promising strategy for the treatment and prevention of bacterial infections. Importantly, its potential is not restricted to infections caused by multidrug-resistant pathogens. Phages can be implemented in situations where the use of antibiotics is limited due to safety, tolerance or specific contradictions for a given patient. This may apply, for example, to pregnancy or people suffering from multiple medical conditions where the range of acceptable agents and doses is limited [7,8]. The clinical efficacy of bacteriophages depends primarily on the transport of virions to the infected niche, their stability in the targeted location, and adaptation to the prevailing physicochemical conditions [9]. Depending on the route of administration, phages encounter various mechanisms of inactivation and removal. In the case of oral administration, these include gastric acidity and accompanying digestive factors [10,11]. Therefore, phage therapy is increasingly based on pharmaceutical technologies that stabilize virions, increase their bioavailability, and enable control of their delivery to the site of infection. Several phage delivery strategies are currently being studied, such as polymeric micro- and nanoparticles, immobilization in hydrogels, as well as lipid carriers, e.g., liposomes and related vesicular systems [10,12,13]. These phage formulations significantly reduce the impact of adverse environmental conditions, ensure good retention in target tissues, and enable controlled local release of phages depending on the properties of the matrix [13].

This review discusses the main mechanisms underlying phage activity, evaluates their therapeutic potential and examines the key factors, particularly those related to the most common delivery routes, that determine the efficacy and safety of bacteriophage therapy. We compare the main strategies currently available for delivering bacteriophages to combat bacterial infection, including topical, oral, pulmonary and systemic infections. The scope of the review also covers the issue of release kinetics at the site of action. The overarching aim is to provide a critical overview of whether bacteriophages, when supported by optimized pharmaceutical and technological delivery strategies, can develop into a practical and effective complement or alternative to conventional antibiotics in combating drug-resistant infections.

## 2. Bacteriophages and Phage Therapy

Bacteriophages, or phages, are viruses that infect bacteria. They represent some of the most abundant biological entities on Earth with an estimated number of 10^31^ virions [14]. Phages are found in virtually all ecosystems, including marine and soil environments, as well as the human gastrointestinal tract. Certain phages can also persist in extreme environments, such as hot springs, glaciers or even outer space. So-called temperate phages can integrate into bacterial genomes and modulate host functions, acting not only as bacterial killers but also as architects of microbial evolution [15].

The antibacterial properties of bacteriophages have been exploited almost since their discovery in the early 20th century. Despite promising beginnings, following the introduction of penicillin and other antibiotics, phage therapy was effectively marginalized in the Western world for several decades [16,17]. Its continuity has been preserved mainly in the former Soviet Union (e.g., in Georgia) and Poland, although it has remained outside the mainstream of medicine [18]. Today, in the face of growing antibiotic resistance, we are seeing a renaissance of interest in phages as a supplement and, in some cases, an alternative to antibiotics. Recent reviews and re-analyses of historical data, along with advances in modern clinical protocols and the combination of phages with antibiotics, highlight both the real potential and the need for standardization and well-designed controlled trials [19,20].

The antibacterial activity of bacteriophages stems from their viral nature, and the interactions between phage and bacterium have direct clinical implications. Phages are distinguished by how they replicate and form a long-term association with the bacterial host. Virulent, or lytic phages, display lytic path and, due to their nature, are quick to reduce bacterial population. In contrast to the virulent counterparts, temperate phages undergo either a lytic or lysogenic cycle. In the lytic cycle, following phage adsorption to bacterial surface receptors and the injection of phage nucleic acid into the cell, replication occurs, ultimately leading to the relatively rapid lysis of the bacterium. On the other hand, the lysogenic cycle is characterized by the integration of viral genetic material into the bacterial chromosome and the co-replication of bacterial and viral DNA. A bacteriophage DNA embedded in the bacterial genome is called a prophage, a key stage in the lysogenic cycle. The choice between lysis and lysogeny depends strongly on host physiology, infection multiplicity, and stress responses. Under certain conditions, prophages may be induced, forcing lysogens into the lytic cycle [18,19,20]. Lysogeny can also reshape bacterial phenotypes through lysogenic conversion—for example, by encoding virulence or antibiotic resistance factors. It may additionally establish superinfection exclusion, which blocks secondary infections. These phenomena have important ecological and clinical consequences [21,22]. Both lytic and temperate phages can mediate horizontal gene transfer through transduction, a process in which phages transfer non-viral DNA from one bacterial host to another. The unintentional spread of antibiotic resistance via phage-mediated transduction continues to raise safety concerns. For phage therapy, screening is routinely performed to select strictly lytic phages with a lack of virulence/antibiotic resistance and reduced transduction capacity [20,22,23,24,25].

Phages offer several unique biological advantages over antibiotics, including high host specificity, allowing them to target pathogens without significantly disrupting the normal microbiota and limiting off-target selection pressure [20,26]. Additionally, they can multiply at the site of infection (auto-dosing), which helps maintain activity at lower doses and adjust the effect to the density of bacteria [27,28]. Equipped with virion enzymes, including depolymerases, they can degrade capsules and biofilm matrices, facilitating penetration and eradication of biofilm populations [29,30]. Their diversity and ability to compose cocktails, as well as evolutionary adaptation, help limit the presence of resistant strains [31]. Importantly, contemporary clinical reviews consistently confirm the favorable safety profile of phages administered by various routes [32,33].

A particularly important phenomenon is a phage-antibiotic synergy. Combined use of phages and antibiotics has been shown to enhance bacterial killing, reduce biofilm formation, and, in some cases, restore susceptibility of resistant strains to conventional drugs [34,35]. This complementary action not only expands therapeutic options but also directly addresses the challenge of antimicrobial resistance (AMR). Phage infection has been demonstrated to disrupt cell envelope integrity and increase membrane permeability, thereby promoting antibiotic entry into bacterial cells [36]. Phage-encoded depolymerases degrade components of the biofilm extracellular matrix, improving antibiotic diffusion and access to embedded bacteria [37]. Furthermore, phages and antibiotics exert selective pressure on distinct bacterial targets and cellular pathways, reducing the likelihood of resistance development by simultaneously attacking different bacterial receptors and metabolic processes [36]. Exposure to sublethal concentrations of certain antibiotics, such as β-lactams or fluoroquinolones, induces bacterial filamentation and inhibits cell division, increasing the cellular surface available for phage adsorption and enhancing phage replication [38]. Certain antibiotics, particularly fluoroquinolones, can also activate the bacterial SOS response via RecA induction, leading to delayed lysis and increased phage production, which further amplifies bacterial killing in phage-antibiotic combinations. This bidirectional interaction can prolong the phage latent period, increase burst size, and ultimately intensify bacterial lysis [39,40,41]. Importantly, phage-driven selective pressure may result in evolutionary trade-offs, whereby the acquisition of phage resistance is accompanied by increased antibiotic susceptibility, effectively lowering the minimum inhibitory concentration required for bacterial eradication [42]. Together, these mechanisms highlight that bacteriophages function not only as direct antibacterial agents but also as biological adjuvants that potentiate antibiotic efficacy and limit the emergence of resistance.

Building on these biological advantages and synergistic interactions, clinical evidence demonstrates the efficacy of phages against MDR pathogens such as *Pseudomonas aeruginosa*, *Acinetobacter baumannii*, *Escherichia coli*, *Klebsiella pneumoniae* and *Staphylococcus aureus*. Successful cases include both life-threatening systemic infections and localized infections of the lungs, bones, wounds or urinary tract [43,44,45,46].

Despite its many advantages, phage therapy also has certain limitations. One of the major challenges is bacterial resistance to phages. Mechanisms include receptor mutations, restriction systems, adsorption-blocking proteins and CRISPR-Cas immunity [47,48]. However, these adaptations often impose evolutionary trade-offs, such as reduced virulence or restored antibiotic susceptibility. This phenomenon, known as phage steering, opens new opportunities to guide bacterial evolution in a way that benefits therapy [20,49,50]. Beyond issues related to bacterial resistance, phage therapy presents additional challenges, including the need for rapid, standardized phage–host matching (“phagograms”) for which clinically validated protocols and turnaround times remain unsettled; this complicates patient selection and cocktail design [51,52]. The host’s immunity can neutralize phages after repeated administration, reducing effectiveness in some cases and complicating retreatment [53]. Genetic safety requires sequencing to exclude lysogeny and transduction potential to limit horizontal gene transfer risks [54,55]. The diversity of regulations and economic constraints related to intellectual property, especially in the case of personalized, dynamically updated cocktails, continues to hinder development, reimbursement and market approval [56,57]. Despite these challenges, phage therapy continues to advance rapidly. Ongoing progress in genomics, standardized manufacturing, and combination strategies with antibiotics or biofilm-targeting agents is steadily translating experimental concepts into practical clinical applications [35,58,59].

Beyond clinical selection, the practical success of phage therapy also depends on robust pharmaceutical development and manufacturing standards. Advancements in good manufacturing practice (GMP) have led to a reduction in variability in the production and quality control processes. However, the implementation of harmonized protocols remains deficient on a global scale [60,61]. The experience gained from the phage therapy naturally gives rise to considerations regarding adjustments to the existing regulatory requirements. It can be hypothesized that a dynamic regulatory framework would favour perception of phage therapy as a platform rather than a discrete biological agent. This may also be relevant to innovative phage formulations. Adaptive standardisation fosters parallel development of multiple phage formulations and enables the tailoring of quality standards to different routes of administration. The proposed procedure would facilitate the pre-approval of the manufacturing process while enabling the monitoring of the process and rapid selection of active phages in the event of bacterial resistance [56,57]. The following recommendations are of key significance: (1) a standardized selection process based on “phagograms”, with predefined schedules and decision thresholds for tailoring phage cocktails to patient needs [51,52]; (2) modular quality documentation covering GMP-compliant manufacturing, purification, sterility and endotoxin testing, as well as genomic safety and stability testing. Appendices for specific phages contain information on identity, host range, and potency [60,61]; (3) phage substitution protocol allowing for phage replacement within the approved composition [56,57]; (4) collection and organization of reference methods and acceptance criteria for post-marketing surveillance, with periodic re-evaluation based on evidence from clinical practice [60,61]; (5) a clear transition from master approval to platform approval after gathering relevant data and evidence on safety and production standardization [56,57].

## 3. Phage Delivery to the Site of Infection

As mentioned earlier, the effectiveness of phage therapy depends not only on the intrinsic lytic activity of the phage but also on the ability to deliver active particles to the site of infection. At the same time, the optimal route of administration remains specific to the infection type. Various models point to the use of more localized and compartment-targeted delivery (topical, aerosol, intraperitoneal, to the body cavity, etc.). For example, for wound infections, topical systems generally achieve better bacterial reduction and tend to outperform systemic delivery for the same infection type, mainly by sustaining high local titer and reduced neutralization [62,63]. As shown in the pulmonary infection model, aerosol delivery dispersed mycobacteriophage throughout the lungs and induced weaker immunity than intravenous delivery of the representative anti-*Mycobacterium tuberculosis* phage Fionnbharth [64,65]. However, rigorous, dose-matched comparisons are scarce.

**Figure 1:**
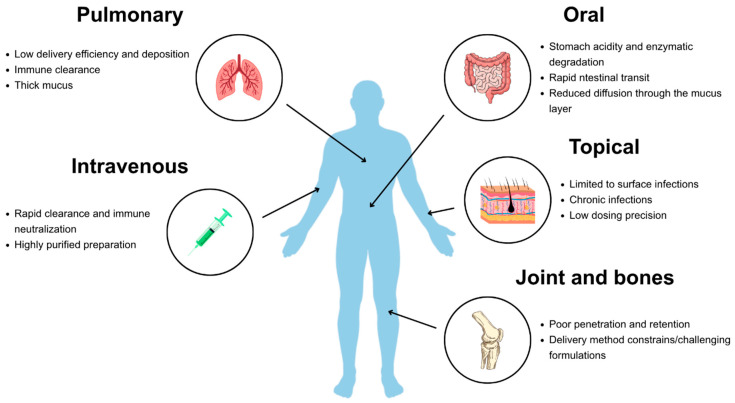
Main limitations encountered during various routes of phage delivery.

Major challenges vary with the route of administration and include rapid clearance by the immune system, enzymatic degradation, and biological barriers that limit bioavailability [66] (Figure 1).

For systemic administration, phages may be neutralized by antibodies, particularly after repeated dosing and eliminated by the reticuloendothelial system [9]. In contrast, surface applications, such as those for chronic wounds or pulmonary infections, face distinct limitations, including reduced penetration into biofilms or inactivation by proteolytic enzymes in the local environment. The acidic pH of the stomach and digestive enzymes represent an additional challenge specific to oral delivery, often impairing efficacy unless protective formulations are employed [66].

To overcome these challenges, a range of administration routes is being explored, including oral, intravenous, inhaled, topical, and localized delivery via hydrogels or implants. Depending on the infection site, phages may be formulated as suspensions, gels, creams, or encapsulated in polymeric carriers to enhance stability and bioavailability [67]. Advances in nanotechnology and biomaterial engineering, particularly in the immobilization and controlled release of biological molecules, further expand the potential to optimize phage delivery.

**Figure 2:**
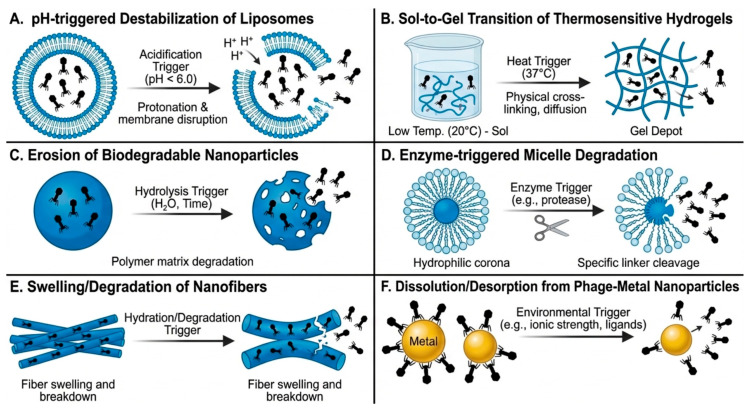
Graphical overview of phage release mechanisms (**A**–**F**) from various carriers.

Once all transport barriers have been overcome, the final step for effective phage delivery is the efficient release from carriers at the site of infection. Bacteriophages are released from liposomes and other nanocarriers through several physicochemical and biological mechanisms [67,68] (Figure 2). These include pH-triggered destabilization of lipid bilayers in inflamed or infected tissues, enzymatic degradation of biodegradable polymeric matrices, fusion of lipid vesicles with bacterial or host cell membranes, and cellular uptake followed by intracellular release [67,68,69].

In particular, for liposomal formulations, phage release is mediated by lipid bilayer destabilization and membrane fusion events or occurs following cellular uptake and intracellular degradation of the carrier. For nanocarrier-based systems that are not inherently stimulus-responsive, phage release is primarily governed by gradual polymer erosion, enzymatic degradation of the carrier matrix, and diffusion-controlled leakage, processes that are enhanced in inflamed, acidic or protease-rich infection microenvironments [70]. In protease-rich or acidic microenvironments typical of infection sites, these processes facilitate localized phage liberation, enabling effective interaction with target bacteria [67].

Ultimately, all these approaches converge on one practical goal: ensuring that active phages reliably reach their target location and remain there long enough to effectively combat infection.

## 4. The Effectiveness and Clinical Insight from Phage Therapy

### 4.1. Oral Administration

Mucosal and gastrointestinal infections represent a major global health burden, often aggravated by antimicrobial resistance. Enteric pathogens such as *Escherichia coli*, *Salmonella* spp., *Clostridium difficile*, and *Campylobacter* spp., as well as biofilm-associated dental pathogens like *Streptococcus mutans*, *Enterococcus faecalis*, *Porphyromonas gingivalis*, and *Fusobacterium nucleatum*, are frequent causes of persistent disease, especially in patients with chronic conditions [71,72]. Their treatment is complicated by polymicrobial interactions, biofilm formation, and reduced antibiotic activity. Such infections remain highly prevalent worldwide and contribute significantly to global mortality rates [71]. In this context, bacteriophage therapy has gained attention as an alternative, with oral administration being particularly relevant for localized gastrointestinal infections.

Importantly, oral phage therapy does not appear to disrupt the intestinal microbiome in either healthy individuals or patients with *Escherichia coli*-associated diarrhoea. Clinical studies indicate that orally administered phages are well-tolerated, safe, and can reduce pathogen load while alleviating diarrheal symptoms, which supports the use of phages as a microbiome-sparing therapeutic strategy against gastrointestinal infections [73,74]. In terms of biocompatibility, oral phage delivery is generally considered safe due to the natural presence of bacteriophages in the human gastrointestinal tract. However, the main limitation of this route of administration is not toxicity, but rapid phage inactivation in harsh gastric conditions, and the studies discussed here primarily focus on improving phage stability and bioavailability rather than on assessment of systemic cytotoxicity or mucosal irritation.

Although oral administration is the most common and convenient route for patients, it poses significant physicochemical challenges for the delivery of bacteriophages in the treatment of gastrointestinal infections [75]. Most free phages are highly sensitive to gastric acidity (pH 1–3) and rapidly lose infectivity without protection [76,77]. Furthermore, the intestinal environment exhibits variable pH (pH 6–7.5 in the small intestine and ~6–7 in the colon), while the mucus layer acts as a particle filter (200–500 nm) and intestinal peristalsis accelerates clearance. These factors underscore the importance of developing immobilized and protected phage formulations to enhance stability, retention, and therapeutic efficacy in the gastrointestinal tract. To overcome these limitations, pH-responsive polymers have been explored, providing both acid resistance and controlled release [78,79].

Free phages are rapidly inactivated under acidic gastric conditions, sometimes losing activity within minutes at pH ~2, which severely limits their oral bioavailability [69,70]. In addition, systemic detection of phages after oral administration is generally low, and the intestinal mucosal membrane further restricts their ability to penetrate and reach target bacteria [80,81]. To overcome these challenges, encapsulation strategies have been developed to protect phages from acidic degradation, improve their survival through the gastrointestinal tract, and facilitate more controlled release at the infection site. Encapsulation allows for producing particles with uniform size and physicochemical properties that do not aggregate upon application, and has become one of the most widely explored approaches for oral phage delivery [75]. Notably, some phages naturally reside in the gut and adhere to mucus, where they may provide a protective effect to epithelial cells against invasive bacteria, which further supports the concept of enhancing phage persistence in the intestinal environment [82].

Among the main polymers used for phage encapsulation, alginate is one of the most extensively studied. Owing to its acid resistance, alginate forms Ca^2+^-crosslinked gels that act as effective diffusion barriers. Release kinetics depend on alginate M/G ratio (M, mannuronic acid/G, guluronic acid), charge density, ion concentration, and layer thickness [78,79]. Modification of the immobilization system, e.g., alginate/κ-carrageenan composites, further improves mechanical stability and swelling control. Chitosan is likewise a frequently employed polymer for phage encapsulation. Layer-by-layer (LbL) chitosan coatings limit proton penetration and enable pH-dependent phage release. Abdelsattar et al. [83] encapsulated *Escherichia coli*-targeting phages in calcium alginate beads (diameter 2.3–2.8 mm) coated with chitosan, while smaller alginate microparticles (50–200 μm) were coated with a copolymer of methacrylic acid and methyl methacrylate, enabling pH-responsive release at pH 2. This carrier system ensured remarkable stability, limiting the titer decline to ~1 log_10_ PFU/mL at pH 2 (compared to complete inactivation of free phage), increasing thermal resistance to 80 °C for 3 min (~0.8–1 vs. ~2.2–2.3 log_10_ reduction), and maintaining the titer without loss for ≥8 weeks at 4 °C. Furthermore, almost all of the encapsulated phages were released within 4–5 h under in vitro intestinal conditions. Importantly, the polymeric carriers used in these formulations (alginate, chitosan) are widely regarded as biocompatible and have a long history of oral pharmaceutical use [83]. Other polymer combinations, such as alginate-chitosan, alginate-carrageenan, or alginate combined with whey protein, have also been explored. It has been established that most tested formulations can protect phages from acidic pH for at least two hours, except the alginate-chitosan combination, where phages become undetectable after one hour [84]. According to Pardo-Freire and Domingo-Calap [85], due to the vast diversity of phages and bacterial hosts, a universal encapsulation method is unlikely; instead, pathogen-specific approaches are required.

To overcome phage acidic degradation during gastrointestinal transit, pH-responsive systems, including Eudragit-based formulations, are being developed to protect phages in the stomach and enable targeted intestinal release [76,86]. Gastric juice-resistant microcapsules composed of the pH-responsive anionic copolymer Eudragit^®®^ S-100 and trehalose were developed to protect bacteriophages Felix O1 from thermal inactivation during spray-drying. The phage-loaded microcapsules were subsequently compressed into solid tablets, resulting in markedly improved stability in simulated gastric fluid (pH 2). In the context of gastrointestinal infections, these pH-responsive polymeric systems enable site-specific phage release in the intestine through polymer dissolution and matrix swelling at near-neutral pH, where pathogenic bacteria are localized. Although direct antibacterial efficacy was not assessed, the formulation demonstrated significant promise for oral phage delivery, suggesting potential in combating gastrointestinal infections caused by *Salmonella* [76,77].

Spray drying is widely used to convert phage suspensions into stable, free-flowing powders. In this process, a solution or suspension is atomized into a stream of heated gas, leading to rapid solvent evaporation and formation of microparticles (typically 1–50 μm). Process parameters can be tuned to achieve powders with low moisture content and high phage viability. Importantly, spray-drying without amorphous stabilizers such as trehalose can result in up to 4-log phage losses, whereas trehalose-containing formulations maintain high viability [87,88].

Because the intestinal mucosal layer represents a major barrier to effective phage delivery, considerable research efforts have focused on developing carriers capable of interacting with and adhering to this protective matrix. Mucoadhesive polymers such as alginate, chitosan, pectin, and carboxymethylcellulose have therefore been investigated as potential excipients [87,88,89,90,91]. For instance, alginate microparticles containing CaCO_3_ and a cocktail of three *Salmonella*-targeting phages demonstrated prolonged retention in the chicken intestine compared to free phages [92].

In addition to polymer-based systems, lipid-based nanocarriers have also attracted attention for their mucoadhesive properties and protective capacity. Among these, liposomal formulations (~300 nm) have shown promise, as their positive charge enhances interaction with the mucosal layer and improves encapsulation efficiency (~50%), leading to prolonged intestinal retention and improved bacterial clearance in vivo. Loh et al. [75] emphasized that lipid-based carriers such as liposomes, transferosomes, and niosomes offer effective protection against enzymatic degradation and immune clearance. Transferosomes are highly deformable phospholipid vesicles containing edge-activating surfactants, which enable them to cross biological barriers such as the mucosa [93]. Niosomes are vesicular systems composed of non-ionic surfactants and cholesterol, characterized by improved chemical stability and lower production costs compared to conventional liposomes [94]. However, the standardisation and scalable manufacturing remain critical for clinical translation.

Similar challenges are observed in the oral cavity, where biofilm-associated dental infections represent a parallel barrier to effective treatment and provide another setting in which innovative phage delivery systems are required. Dental infections, particularly biofilm-associated conditions such as periodontitis, peri-implantitis, and recurrent apical periodontitis, present challenges analogous to gastrointestinal infections. *Enterococcus faecalis* is a primary pathogen in persistent root canal infections, where its survival in complex canal structures often renders conventional endodontic treatments ineffective [72]. Standard agents like calcium hydroxide or sodium hypochlorite fail to fully eradicate these bacteria, particularly in high-pH or biofilm-protected environments. Bacteriophage therapy offers a highly specific antibacterial alternative. In addition to whole phages, phage-derived lysins represent a distinct strategy, capable of lysing *Enterococcus faecalis* even within biofilms. To overcome enzymatic instability in vivo, advanced delivery systems such as metal–organic frameworks (e.g., ZIF-8) have been employed. Encapsulation of lysins like LysPd138 within ZIF-8 enables pH-responsive, sustained release, protects enzymatic activity, and promotes osteogenesis via Zn^2+^ ion release. In vitro, ex vivo, and in vivo studies demonstrate that such systems effectively eradicate *Enterococcus faecalis* biofilms and support bone regeneration, addressing both microbial clearance and tissue repair [95].

Collectively, these findings highlight the versatility of phage-based strategies in tackling biofilm-associated and multidrug-resistant infections across different mucosal sites. By integrating targeted antimicrobial activity with engineered delivery platforms, phages present a promising adjunct or alternative to conventional antibiotics in both gastrointestinal and dental applications, supporting more effective and microbiome-friendly therapeutic outcomes [95]. As presented in Table 1, recent in vivo studies have provided significant evidence confirming the efficacy of phages distributed via the oral route using distinct formulations. 

### 4.2. Intravenous Administration

Systemic infections such as sepsis and bacteremia caused by multidrug-resistant pathogens remain a major clinical threat. Frequent culprits include *Escherichia coli*, *Klebsiella pneumoniae*, *Pseudomonas aeruginosa*, *Acinetobacter baumannii* and *Staphylococcus aureus*, with ESBL- and carbapenem-resistant strains severely limiting treatment options and driving high mortality [104]. Intravenous phage therapy offers direct access to the bloodstream and systemic distribution, but its efficacy is challenged by immune clearance and neutralization [105]. Consequently, optimizing delivery strategies is essential for successful clinical translation.

For the treatment of systemic or severe infections, intravenous administration is preferred, as it is considered safe and follows general principles of drug administration [104]. Importantly, intravenous delivery of bacteriophages has already been successfully applied in clinical settings (Table 2). For example, Cano et al. [106] reported a case of a 62-year-old patient with recurrent knee prosthesis infection facing amputation, who received phage therapy combined with oral minocycline for 40 days, resulting in clinical improvement and a symptom-free period of 34 weeks.

Intravenously administered bacteriophages are generally well tolerated, as evidenced by clinical case reports and compassionate-use studies (Table 2). Reported adverse effects are typically mild and transient, such as temporary elevations in liver enzymes, with no consistent evidence of severe systemic toxicity.

However, intravenous phage administration may potentially cause adverse effects. For instance, a 72-year-old patient with chronic methicillin-resistant *Staphylococcus aureus* (MRSA) prosthetic joint infection discontinued phage therapy after three days due to a transient increase in transaminase levels, suggesting reversible liver injury [107]. Nevertheless, the studies show that following intravenous administration, phages must rapidly reach the bacterial host; otherwise, their titers in the bloodstream drop sharply, reducing therapeutic efficacy. Phages primarily accumulate in specific organs like the liver, spleen, lymph nodes, lungs and muscles [108]. A major limitation is also the presence of neutralizing antibodies in the bloodstream, which may compromise therapeutic effectiveness, although their precise role remains unclear [109,110]. Evidence supporting this ambiguity comes from a case study of a 15-year-old patient with disseminated *Mycobacterium abscessus* infection, who received a phage cocktail for 32 weeks and showed no evidence of phage neutralization despite the detection of such antibodies [35]. These challenges have stimulated interest in alternative delivery systems aimed at improving phage stability and therapeutic performance.

Among these, liposomes composed of a phospholipid bilayer have been widely explored due to two main advantages: (I) mitigating adverse effects and (II) enhancing phage activity while reducing inactivation mechanisms [111]. Encapsulation of phages in cationic liposomes has been shown to protect them from neutralizing antibodies, thereby preserving their antibacterial efficacy [68,112]. Importantly, when administered intravenously, phages can be phagocytosed by macrophages, which influences their bioavailability and distribution. Liposomal encapsulation, therefore aims not only to extend circulation time and stability but also to mitigate rapid clearance by the immune system [113]. From a chemical perspective, liposomes protect phages against environmental stress and provide physical separation from inactivating interfaces. Their composition determines both encapsulation efficiency and tissue behaviour. Typical preparations include saturated phosphatidylcholine and cholesterol to stabilize the bilayer, anionic lipids such as DSPG-Na to modulate surface charge, and PEGylated lipids such as DSPE-PEG to reduce unwanted interactions with host cells. Incorporation of polyethylene glycol (PEG) imparts “stealth” properties, prevents aggregation and opsonization, decreases phagocytic uptake, and extends circulation time [114]. Moreover, liposomes can reduce epithelial uptake, which is advantageous for extracellular infections, and they tolerate nebulization well after the addition of mannitol or sucrose to the aqueous phase [115]. From a safety perspective, liposomal phage formulations were consistently reported as biocompatible in both animal models and clinical case studies. No acute toxicity, severe immune reactions, or carrier-related adverse events were observed following intravenous administration of liposome-encapsulated phages, suggesting that encapsulation does not compromise the favorable safety profile of bacteriophages [108,110,111,112,113,114].

Nanodrug delivery systems also enable controlled release of bioactive agents, thereby enhancing their half-life. For instance, studies by Singla et al. [68,112] demonstrated that bacteriophages encapsulated in nanoparticles exhibited prolonged circulation in the bloodstream compared to free phages, which were rapidly cleared by the mononuclear phagocyte system (MPS). Similarly, Chadha et al. [115] reported that a cocktail of phages targeting *Klebsiella pneumoniae*, conjugated with cationic liposomes, showed extended residence time in both blood and organs. However, despite the rapid development of nanotechnology and numerous reviews published between 2018 and 2025, there is still a lack of new, comparative in vivo studies evaluating the pharmacokinetics of intravenously administered phages encapsulated in nanocarriers. Current knowledge supporting the use of stealth strategies such as PEGylation is largely extrapolated from non-phage systems or older experimental models. As a result, classical in vitro studies remain the most robust evidence base in this field [68,112,115].

**Table 2:** The examples of phage therapy using intravenous delivery.

Formulation	Phage	Infection	Model	Treatment Mode	Outcome	References
Liquid phage suspension	Phage cocktail AB-SA01	*Staphylococcus aureus*	Human clinical trial	Phages (3 × 10^9^ PFU/mL) administered twice daily for 14 days	Safe, no adverse reactions. No phage resistance. The efficacy was not the primary endpoint	[116]
	Phage cocktail (EFgrKN and EFgrNG)	Vancomycin-resistant *Enterococcus faecium*	Human case report, VRE in liver transplant patient	Phage (8.1 × 10^7^ PFU/mL and 5.2 × 10^8^ PFU/mL) administered daily for 20 days.	Safe, clinical improvement, no adverse events	[117]
	Phage cocktail (HP3, HP3.1, ES17 and ES19)	ESBL *Escherichia coli*	Human case report, prostate and urinary tract infection	Phage (3 × 10^10^ PFU/mL) administered twice daily for 14 days	Safe, symptom resolution, no adverse events	[118]
	Phage ΦAb4B	*Acinetobacter baumannii*	Mice bacteraemia model	Once daily (10^9^ PFU/mL) for 7 days	67% survival with phage alone, 91% with phage-ciprofloxacin, no acute toxicity	[119]
	Phage cocktail (BPsΔ33HTH_HRM10, Muddy and ZoeJΔ45)	*Mycobacterium* *abscessus*	Human case report	Phages administered (10^9^ PFU/mL) twice daily for ≥6 months	Clinical improvement in lung function and symptoms; one case was limited by neutralizing antibodies	[120]
	Phage KpJH46Φ2	*Klebsiella* *pneumoniae*	Human case report of prosthetic knee infection	40 doses of phage (6.3 × 10^10^ PFU/mL) and minocycline	Resolution of infection, recovery of function, and no adverse effects	[106]
	Cocktail of phages (phiCDHM1, phiCDHM2, phiCDHM5 and phiCDHM6)	*Clostridium difficile*	Hamster model of acute infection	0.8 mL of single phage or phage cocktail (10^8^ PFU/mL) every 8 h until 36 h	The phage cocktail prolonged the time to onset of severe disease by ~33 h	[102]
	Phage PASA16	*Pseudomonas* *aeruginosa*	Human case report of chronic bone infection	Phage (1.72 × 10^11^ PFU/mL) and ceftazidime administered twice daily for 14 days	Good clinical outcome in >80% patients; minimal side effects	[121]
	Phage DS6A	*Mycobacterium* *tuberculosis*	Humanized mice model of pulmonary infection	10 doses of phage (10^11^ PFU/mL)	Reduced bacterial load, improved pulmonary function, and increased body weight	[122]
	Cocktail of phages	*Escherichia coli*, *Enterococcus* *faecium*, *Staphylococcus* *aureus*, *Klebsiella* *pneumoniae*, *Klebsiella* *aerogenes*, *Pseudomonas* *aeruginosa*, *Enterobacter* *cloacae*	Human case report, AMR infections	Phage application (10^9^ PFU/mL) every 12 h for 14 days	Good clinical outcome in 66% cases, no major adverse reactions.	[123]
	Phage AB-PA01	*Pseudomonas* *aeruginosa*	Human case report, chronic respiratory infection	Phage application (4 × 10^9^ PFU/mL) every 6 h for 8 weeks	Good clinical resolution from pneumonia; no further CF pulmonary exacerbation for 3 months; no adverse events	[124]

### 4.3. Administration in Contact with Bone and Joints

Bone and joint infections, including osteomyelitis, prosthetic joint infections, and septic arthritis, are difficult to treat due to biofilm formation and poor antibiotic penetration into bone tissue. These infections, often caused by multidrug-resistant bacteria such as *Staphylococcus aureus*, *Enterococcus* spp., and Gram-negative pathogens, frequently lead to chronic disease and repeated surgeries [125]. In such cases, bacteriophage therapy represents a promising alternative, particularly when administered locally to deliver high concentrations directly to the site of infection [126,127].

To effectively combat infections, bacteriophages must locate their bacterial hosts as quickly as possible. For this reason, it is generally recommended that their administration take place as close as possible to the infection site [67]. Local delivery not only accelerates phage–host interactions but also enables immediate phage replication at the target site. Noteworthy is that, in some cases, combining local formulations with intravenous injection has been proposed to further sustain therapeutic concentrations and counteract systemic clearance, thereby improving treatment efficacy [128].

Table 3 summarizes the examples of in vivo trials on the phages targeting bone and joint infections using various delivery approaches. One promising strategy for local delivery is the use of hydrogels, three-dimensional (3D) networks of hydrophilic polymers that act as reservoirs for therapeutic agents [129]. One of the main advantages of hydrogels is that they can be engineered to control phage release kinetics. At the site of infection, phage release from hydrogels is governed by temperature-induced gel relaxation, gradual diffusion through the hydrated polymer network, and enzymatic degradation of the matrix in protease-rich inflammatory environments. For instance, poloxamer 407 (P407), which undergoes reversible temperature-dependent gelation, enables both rapid initial release and subsequent sustained diffusion of phages. This approach has been shown to reduce bacterial biomass in multidrug-resistant *Acinetobacter baumannii* models [130]. From a chemical perspective, key factors influencing hydrogel performance include copolymer concentration, cross-linking modifications, ionization, and the addition of protein–polysaccharide components such as alginate-modified gelatin, which regulate porosity and charge [130,131,132]. Several studies highlight the therapeutic potential of hydrogel-based phage delivery. Barros et al. [133] developed an alginate-nanohydroxyapatite hydrogel capable of encapsulating phages, ensuring their prolonged release, as 97% of phages were released after 24 h. After loading into the hydrogel, phage activity both in vitro and ex vivo increased, and the osteogenic properties of the hydrogel were maintained. Similarly, Wroe et al. [134] created hydrogels with adhesion peptides for encapsulating phages against *Pseudomonas aeruginosa*. In a mouse model of bone infection, phages immobilized in the hydrogel reduced *Pseudomonas aeruginosa* infection more effectively than free phages. These hydrogels were based on a four-arm poly(ethylene glycol)-maleimide (PEG-4MAL) macromer, crosslinked via thiol-maleimide chemistry with cysteine-containing, protease-degradable peptides. The modular design enabled incorporation of adhesion motifs (e.g., RGD, GFOGER), which are derived from extracellular matrix proteins and are specifically recognized by cell-surface integrins such as αvβ3 and α5β1 for RGD, and collagen-binding integrins, including α2β1 for GFOGER. Furthermore, the alteration of the peptide crosslinkers allowed for controlled phage release in protease-rich environments such as infected bone tissue [134]. Biocompatibility assessment revealed no adverse tissue reactions, with reduced local inflammation and preserved osteointegration in the evaluated models. Histological analyses confirmed good tissue compatibility of the hydrogel-phage systems, supporting their suitability for local application in bone infections, although standardized comparative studies remain limited [130,131,132,133,134]. Clinical studies further support this strategy. Ferry et al. [135] successfully treated knee infections caused by MRSA by applying phage-loaded hydrogels directly onto infected prostheses. The hydrogel used was DAC^®^ (Defensive Antibacterial Coating), a commercial system composed of two biodegradable polymers: hyaluronic acid and polylactic acid (PLA). Phages (PP1493 and PP1815) were suspended in physiological buffer (DPBS or water for injection, according to the manufacturer’s instructions), and it was demonstrated that, once mixed with the DAC^®^ hydrogel, they were released rapidly in a burst fashion, while maintaining stable titers (plaque-forming units per millilitre, PFU/mL) for at least 6 h. In another approach, Ismail et al. [136] enhanced phage retention on prosthetic material by coating tricalcium phosphate (Ca_3_(PO_4_)_2_) with calcium alginate hydrogel. The hydrogel layer served as a biocompatible matrix for phage immobilization, protecting phages from rapid washout and environmental inactivation while enabling their gradual release from the implant surface. This strategy resulted in prolonged local phage availability and improved lytic activity against bacteria associated with implant-related infections. More recently, Chen et al. [137] designed a hydrogel combining bacteriophage cocktails with vancomycin to treat fracture-related infections (FRI) caused by MRSA. The hydrogel used as a phase vehicle in this study was based on aqueous dispersions of sodium carboxymethyl cellulose (CMC, Sigma-Aldrich, USA). This formulation significantly reduced biofilm mass (by 99.72% in vitro), lowered bacterial load in vivo, and preserved phage activity for up to eight days, demonstrating the synergistic potential of combining phage therapy with antibiotics. In general, hydrogel-based delivery systems for bone and joint infections were generally well tolerated in the reported studies. Cytotoxicity testing and in vivo evaluations indicated minimal local inflammatory responses, preserved tissue compatibility, and, where assessed, maintenance of osteogenic properties. Importantly, the use of biodegradable and clinically established polymers limited systemic exposure, supporting the suitability of these carriers for local administration.

Beyond hydrogels, protein-polymer carriers also show promise. Xu et al. [138] reported the use of silk fibroin microparticles modified with cationic polyethyleneimine (PEI), which conferred a positive surface charge enabling electrostatic adsorption of anionic phages. These microparticles achieved high phage loading (~1.25 × 10^10^ PFU/mg), acted as local reservoirs, and improved therapeutic efficacy in a mouse model of MRSA infection for up to 72 h. Notably, this study represents one of the few examples in which biocompatibility was quantitatively assessed, demonstrating low hemolytic activity and good cellular compatibility, thereby strengthening the translational relevance of this carrier system.

Finally, additive manufacturing techniques are opening new avenues for phage-based therapies. Bouchart et al. [139] developed ceramic pellets containing a cocktail of phages against *Staphylococcus aureus* and *Escherichia coli* to reduce biofilm formation on implants. The authors demonstrated that the 3D-printed bioceramic scaffold served not only as a structural bone substitute but also as a local delivery system for bacteriophages. In vitro experiments confirmed that the embedded phages remained viable after the printing process and were gradually released over several days, effectively inhibiting bacterial growth and biofilm development. This approach highlights the potential of combining additive manufacturing with phage therapy to create personalized anti-infective bone implants, addressing the challenge of antibiotic-resistant pathogens in orthopedic surgery.

**Table 3:** The examples of phage therapy through delivery to the bone and joints.

Formulation	Phage	Infection	Model	Treatment Mode	Outcome	References
Liquid phage suspension	Phages Sb-1 and PAT14	Methicillin-resistant *Staphylococcus aureus* and *Pseudomonas aeruginosa*	Rat model of implant-related infection	Local injection of 0.1 mL of phage suspension (10^7^ PFU/mL) in combination with antibiotics	Significant reduction in bacterial load and biofilm disruption	[140]
	Cocktail of seven virulent phages	Methicillin-resistant *Staphylococcus aureus*	Rabbit model of osteomyelitis	Injection of 15 µL of phage cocktail (10^12^ PFU/mL) into the infected soft tissues	Improved general condition of animals, significant reduction in inflammation and necrosis	[141]
	Phage ISP	Methicillin-resistant *Staphylococcus aureus*	Sheep model of fracture-related infection (FRI)	Phage (10^8^ PFU/mL) local administration 3 times/day for 10 days	Well-tolerated administration, rapid phage clearance and neutralization	[67]
	Phage cocktail BFC1 (phage ISP, PNM and 14/1)	*Staphylococcus aureus*	Human case study, polymicrobial pelvic bone allograft chronic infection	Intraoperative administration of 50 mL of phage cocktail BFC1 (10^7^ PFU/mL) to the infected site via drainage	Clinical/microbiological improvement, but recurrence if not all pathogens are covered	[142]
	Phage Pa53	*Pseudomonas aeruginosa*	Human case study, chronic hip prosthesis infection	10 mL of phage on the first day, then 5 mL via joint drainage for 2 weeks in association with antibiotic	Effective eradication of infection in combined treatment with meropenem, with no severe adverse effects	[143]
Phage-loaded hydrogel	CRISPR-Cas9 modified phage loaded on alginate hydrogel	*Staphylococcus aureus*	Rat model of osteomyelitis and soft tissue infection	Phage loaded hydrogels (10^7^ PFU/mL) injected (100 µL) into the defect space	Reduced soft tissue infection, limited bone effect	[144]
	Phage IPS loaded in carboxylmethylcellulose (CMC) hydrogel	*Staphylococcus* *aureus*	Rabbit model of fracture-related infection (FRI)	Prevention and treatment setting, subcutaneous injections (10^8^ PFU/mL) and phage loaded hydrogels (10^9^ PFU/mL)	Phage in saline was effective in prophylactic mode. Phage immobilization limits the exposure to neutralizing antibodies, with no statistically significant reduction in the bacterial load	[145]
	Phages ΦPaer4, ΦPaer14, ΦPaer22, and ΦW2005A loaded in PEG-4MAL hydrogel crosslinked with BPM-2	*Pseudomonas* *aeruginosa*	Mice radial defect model	3 µL of hydrogel with phages (1.2 × 10^8^ PFU/mL each) loaded into the defected space	4.7-fold reduction in bacterial load at 7 days postimplantation, and lowered inflammation	[134]
Implants	Orthopaedic K-wires coated with Phage, linezolid and hydroxypropyl methylcellulose gel	*Staphylococcus* *aureus*	Mice model of prosthetic joint infection	Different coating variants (e.g., phage 10^9^ PFU/mL phage mixed with HMPC and 5% w/w linezolid) were surgically placed into the joint, phage	Reduction in bacterial adherence, limited inflammation, and faster resumption of locomotion	[146]
	Phage vB_SepM_Alex	*Staphylococcus* *epidermis*	Rat model of prosthetic joint infection	Intra-articular injections of phage (10^8^ PFU/mL) in 5 days post-implantation	Preliminary set of pharmacokinetics: maximum phage concentration after 2 h and mean residence time of ~3 h	[147]
	Silver-coated plate with DAC^®®^ gel loaded with Intesti phage cocktail	Multi-drug resistant Gram-negatives, *Staphylococcus aureus*	Human case study, fracture-related infection	Surgical placement of an implant in the bone defect	Infection control, good bone healing, sustained phage release	[148]
Liposomes	Liposome-phage Sb-1 nanoconjugates (Lip@Phage)	Methicillin-resistant *Staphylococcus aureus*	Rat prosthetic joint infection model	local application (100 μL) twice daily for a duration of 14 days	Effective reduction in bacterial load, improved osteomyelitis recovery	[149]

### 4.4. Aerosols and Inhalation in the Treatment of Pulmonary Infections

Pulmonary infections, particularly in patients with chronic lung diseases such as cystic fibrosis, bronchiectasis, or chronic obstructive pulmonary disease (COPD), are often caused by multidrug-resistant bacteria, including *Pseudomonas aeruginosa*, *Staphylococcus aureus* (MRSA), *Klebsiella pneumoniae* and *Acinetobacter baumannii*. Biofilm formation within the respiratory tract further complicates treatment by limiting antibiotic penetration and promoting persistent, recurrent infections [150]. Inhaled phage therapy offers a promising strategy by achieving high local concentrations at the site of infection while minimizing systemic exposure [151]. This is demonstrated by the results of various in vivo trials (Table 4).

Acute respiratory infections affect both the upper and lower respiratory tracts, with pneumonia ranking as the fourth leading cause of death worldwide [119,152]. Tuberculosis, the most significant bacterial lung infection, requires prolonged antibiotic therapy that fosters resistance development, while cystic fibrosis (CF) predisposes patients to recurrent pulmonary infections due to thick mucus that promotes bacterial biofilm formation and complicates antibiotic treatment [153]. Phage therapy has shown considerable potential in managing respiratory infections, particularly when stabilized phage formulations are prepared as powders through lyophilization. Lyophilization, a process of dehydrating phage-containing liquids, not only prevents phage inactivation during rehydration but also ensures long-term stability of phage preparations [75]. Importantly, these dry powder formulations are also convenient for administration in respiratory infections. For example, Prazak et al. [154] developed a phage nebulization system for potential application in mechanically ventilated patients with *Staphylococcus aureus-induced* pneumonia, demonstrating improved survival in infected rats. Their findings suggest that nebulization enhances phage concentration in the lungs, thereby increasing therapeutic activity. Similarly, Guillon et al. [155] showed the effectiveness of phage nebulization against *Pseudomonas aeruginosa* in a porcine model, using a static-mesh nebulizer to aerosolize a phage cocktail suspended in sodium chloride solution. To further increase therapy effectiveness, liposome-based delivery systems have been developed. Singla et al. [68] used cationic liposomes to deliver phages against *Klebsiella pneumoniae*, achieving 94.6% elimination of intracellular bacteria. Likewise, Nieth et al. [156] developed anionic giant unilamellar liposomes targeted at mycobacteria, which proved more effective in penetrating host cells than free phages. The liposomes consisted of a mixture of DOPS/DOPC with an admixture of dye (50:49.75:0.25 mol% DOPS:DOPC:TexasRed-DHPE) and were produced by rehydration of the lipid film and extrusion (1 µm pores) for encapsulation of phage TM4. The authors also employed gel-assisted swelling and the reverse emulsion method to improve encapsulation efficiency. For inhalation therapy against *Pseudomonas aeruginosa*, Sawant et al. [114] formulated smaller nanoliposomes composed of HSPC:DSPG-Na:DSPE-PEG:cholesterol (3:2; 0.5; 1.7), with a particle size of ~170 nm, a zeta potential of ~ −50 mV, and an encapsulation efficiency of ~58%. These liposomes reduced epithelial uptake, which is advantageous for extracellular infections, and tolerated nebulization well when mannitol or sucrose was added to the aqueous phase. These findings highlight how lipid ratios, charge-modifying components, and PEGylated lipids critically influence particle size, zeta potential, encapsulation efficiency, and stability during aerosolization.

Regarding safety and biocompatibility, inhaled phage formulations and aerosolized delivery systems were generally well tolerated in the reported in vivo studies. No significant pulmonary toxicity, excessive inflammatory responses, or adverse effects on lung tissue architecture were observed, while systemic exposure remained limited due to localized administration. Nevertheless, formulation composition, particle size, and aerosolization method seems to remain critical factors influencing respiratory safety and deposition efficiency.

Beyond liposomal formulations, other nanocarrier systems are also being investigated for pulmonary infections, particularly in CF, where mucus accumulation and biofilms present additional challenges. Gondil et al. [157] reported that chitosan nanoparticles adhered strongly to mucus and were successfully used to encapsulate a phage-derived lysin targeting *Streptococcus pneumoniae*. Similarly, Agarwal et al. [158] developed polylactic-co-glycolic acid (PLGA) microspheres that enabled efficient delivery of phages to the lungs, reduced *Pseudomonas aeruginosa* biofilms, and sustained high phage titers through in situ replication. Another innovation came from Cinquerrui et al. [159], who employed microfluidic technology to overcome the reduction in phage titers caused by isopropanol used in conventional liposome preparation. This approach minimized solvent exposure, thereby improving encapsulation efficiency and preserving phage activity. Importantly, available preclinical studies indicate that inhalation of phage formulations does not induce lung inflammation or histopathological damage in the evaluated models. Several studies explicitly reported the absence of elevated pro-inflammatory cytokines, confirming good pulmonary biocompatibility of both liquid and dry powder phage formulations, although most data are derived from preclinical studies [154,155,156,157,158].

At the same time, dry phage formulations for pulmonary delivery are being intensively developed, with particular focus on enhancing encapsulation efficiency and enabling efficient penetration into the alveoli (Table 4). A novel and increasingly targeted strategy in the fight against respiratory infections is the direct inhalation of phages, which allows for localized delivery to the lungs and has the potential to reduce inflammation and bacterial load without disrupting the resident microbiome [160]. Recent studies emphasize the importance of formulation chemistry, including the use of stabilizing excipients such as sugars, amino acids, or polymers, which protect viral particles during spray-drying or freeze-drying processes. These advances not only improve phage stability under ambient conditions but also facilitate controlled aerosolization and deposition in the lower respiratory tract, thereby significantly broadening the clinical applicability of inhaled phage therapy [161]. Overall, advances in formulation chemistry have enabled inhaled phage therapies that combine effective pulmonary delivery with safety and tolerability profiles.

**Table 4:** The examples of phage therapy using pulmonary delivery.

Formulation	Phage	Infection	Model	Treatment Mode	Outcome	References
Liquid aerosol	D29 mycobacteriophage	*Mycobacterium* *tuberculosis*	Mice lung infection model	Endotracheal or nose-only inhalation of phage aerosol	Effective phage deposition in lungs (10% of phage particles), no lung inflammation	[162]
	D29 mycobacteriophage	*Mycobacterium* *tuberculosis*	Mice lung infection model	Nose-only inhalation of phage aerosol	Significant reduction in bacterial burden with prophylactic delivery of phage	[163]
	Phage cocktail (2003, 2002, 3A, and phage K)	Methicillin-resistant *Staphylococcus aureus* clinical isolate AW7	Rat model pneumonia	Single application of a nebulized phage cocktail at a concentration of 10^10^ or 10^11^ PFU/mL	Reduced lung bacterial burden, improved survival of infected rats (50%)	[154]
	Cocktail of two phages (Eliava Institute, Tbilisi)	*Achromobacter* *xylosoxidans*	Human case study, Cystic fibrosis and chronic infection	Inhalation of phage (3 × 10^8^ PFU/mL) once daily and orally twice daily, for 20 days. The treatment course was repeated a total of 4 times: at 1 month, 3 months, 6 months and 12 months after initial treatment	Increased lung function, reduced cough and dyspnea	[164]
	Personalized single-phage preparation	Carbapenem-resistant *Acinetobacter baumanii*	Chronic obstructive pulmonary disease and infection, case study	Inhalation of phage every 12 h for 13 days	Clearance of the infection	[165]
	Phage cocktail (JW Delta, JWT, 2-1)	*Achromobacter* *xylosoxidans*	Lung-transplant CF patient with infection, case study	Phage nebulization three times a day in two rounds	Improved lung function, no side effects, no recolonization in two years after phage therapy	[166]
	Phage cocktail (PP1450, PP1777 and PP1902)	*Pseudomonas aeruginosa*	Pneumonia model in piglets	2 and 11 h after bacterial challenge, application of a phage cocktail of equal titres of ~1.1 × 10^10^ PFU/mL by inhalation	High phage concentration in the lungs, rapid reduction in bacterial density	[155]
	Phage KPP10 + ceftazidime/avibactam + CaEDTA	*Pseudomonas* *aeruginosa*	Mice model of chronic lung infection,	Intranasal inhalation of phage (2 ×10^7^ PFU/mL), in combination with CaEDTA and ceftazidime/avibactam	Cleared bacteria in the lungs, reduced expression of genes related to chronic infections	[167]
	Phage vB_AbaM_Acibel004	*Acinetobacter* *baumanii*	Mice pneumonia model	Single application of 25 µL phages (5 × 10^6^ PFU/mL) by intratracheal aerosolization following orotracheal intubation under isoflurane anesthesia	Lower bacterial counts in lungs, lack of inflammatory and adverse effects	[168]
	Phage PEV31	MDR *Pseudomonas aeruginosa* clinical isolate	Mice model pulmonary infection	Doses of 10^7^ and 10^9^ PFU by the intratracheal route	Reduced lung bacterial load by 2-log_10_ suppressed proinflammatory cytokines	[169]
	Phage PEV31	MDR *Pseudomonas aeruginosa* clinical isolate	Mice model pulmonary infection	Doses of 7.5 × 10^4^, 5 × 10^6^, and 5 × 10^8^ PFU by the intratracheal route	Reduced bacterial load by 1.3–1.9 log_10_, dose-dependent effect of phage therapy	[170]
Dry powder aerosol	Lyophilized phage-loaded PLGA microparticles	*MDR Pseudomonas aeruginosa*	Pulmonary delivery of 1 mg of dry-powder phage-microparticles	Mice with cystic fibrosis	Reduced bacterial counts and 100% rescue from pneumonia-associated death	[158]
	Phage PEV20 spray-dried with lactose and leucine	MDR *Pseudomonas aeruginosa*	Intratracheal administration of 1–4 mg of dry-powder phage PEV (2 × 10^7^ PFU/mg)	Mice lung infection model	reduced bacterial load (~2 log)	[171]
	Spray-dried PEV20 with ciprofloxacin	MDR *Pseudomonas aeruginosa*	Intratracheal administration of powders (1 mg) of single ciprofloxacin (0.33 mg), single PEV20 (10^6^ PFU/mg) and the combination	Mice model with acute lung infection	Significant reduction in bacterial load (~ 6 log_10_) using the combination of PEV20 and ciprofloxacin	[172]

### 4.5. Topical Administration

Skin and soft tissue infections, which includes infected burns, wound infections, and ulcers, are often complicated by antibiotic resistance. The most common pathogens responsible for skin infections include *Staphylococcusaureus*, *Pseudomonas aeruginosa*, *Streptococcus* spp., *Enterococcus* spp., *Klebsiella pneumoniae*, and *Escherichia coli* [173]. In patients with diabetes, skin infections occur more frequently and are more difficult to treat [174]. Moreover, biofilms present in over 78% of chronic wounds further complicate therapy. In severe cases, the infection may progress to sepsis, which is a life-threatening condition [175]. In this context, phage therapy represents a promising alternative, and numerous studies have evaluated the use of phage therapy via topical administration. Literature on this topic is summarized in Table 5, with an emphasis on the in vivo investigation with different delivery strategies.

Several systems for topical phage delivery exist, such as ointments, creams, gels, as well as balms and suspensions. Other carriers, such as hydrogels, liposomes, nanoemulsions, adhesives, and films, can also be used. When applied topically, phage formulations should be tailored to the treatment site—they must be minimally irritating, user-friendly, removable without difficulty, and stable to reduce frequent reapplication. Importantly, the carrier itself should preferably provide bacteriostatic activity. Beyond these requirements, formulations containing lytic phages must preserve structural integrity and viability during storage, ideally protected from light at 4 °C [176].

Efforts to improve phage efficacy in topical administration include the use of cationic liposomes (~200 nm), which stabilize phages and protect them from degradation. In animal models, Chhibber et al. [111] demonstrated that phage-loaded liposomes used to treat MRSA-induced wound infections led to greater phage accumulation at the site of infection, resulting in faster bacterial clearance and improved wound healing. Similarly, Chadha et al. [115] demonstrated that a liposome-encapsulated phage cocktail against *Klebsiella pneumoniae* significantly reduced bacterial burden in burn wound infections, accelerated infection resolution, and prevented mortality-even when treatment was initiated late. Another innovative approach involves polymeric nanospheres that release phages in response to temperature changes. The developed structures, composed of a temperature-sensitive polymer, reduce their volume above 34 °C, leading to controlled phage release and effective elimination of *Staphylococcus aureus* [93]. Furthermore, alternatives to liposomes, such as transferosomes, which are liposomes containing detergents, should also be explored. Due to the presence of edge-activating surfactants, transferosomes exhibit high membrane flexibility that enable penetration through narrow intercellular junctions and facilitate transport across the skin. Transferosomes have been used in phage therapy for skin and soft tissue infections caused by *Staphylococcus aureus* in a mouse model. These carriers showed better skin penetration and higher levels of soft tissue protection than a cocktail of free phages [93,177].

Due to the necessity of maintaining phages at the infection site for prolonged periods, hydrogels are also gaining increasing importance in the treatment of wound infections. They are particularly attractive because they create a moist environment that promotes wound healing and limits pathogen proliferation [178]. Furthermore, they exhibit flexibility, plasticity, and biocompatibility [179]. Hydrogels are widely used for phage delivery, both alone and in combination with other active substances such as non-ionic polymers, including polyethylene glycol (PEG) and polypropylene glycol (PPG), which have been applied in the treatment of skin infections [180]. Among the studied formulations is a sodium alginate-based hydrogel that enables the encapsulation of phages targeting *Staphylococcus aureus*. It was demonstrated that phages retain their stability in the hydrogel for at least 28 days, and their application in mice resulted in effective control of bacterial infections and accelerated skin regeneration [181]. Similarly, 3D-printed alginate-calcium chloride hydrogels containing phages against *Escherichia coli* enabled gradual phage release over 24 h, although encapsulation was found to limit activity by delaying phage diffusion through the hydrogel matrix [182]. In another study, thermosensitive hydrogel dressings for wounds were investigated as they prevent further damage of the wound. Yan et al. [130] assessed the effectiveness of a thermosensitive hydrogel containing phages in treating MDR *Acinetobacter baumannii* wound infections on pig skin models. In vitro and ex vivo studies showed that the poloxamer 407 hydrogel stabilizes phages and allows their gradual release, maintaining a high concentration at the infection site. These findings suggest that the phage hydrogel P407 may be an effective treatment for wounds infected with MDR *Acinetobacter baumannii*. Moreover, thermosensitive dressings were shown to enable continuous phage release at the wound site and improve antibacterial activity compared to the use of free phage suspensions. Additionally, thermosensitive hydrogel dressings containing phages for wound treatment can target biofilms, making them a promising therapeutic option for chronic wound infections [183]. In the study by Narayanan et al. [184], hydrogel wound dressings containing phages against *Escherichia coli* were developed, using carrageenan as a carrier to stabilize the phages, effectively inhibiting bacterial growth and preventing resistance development. Importantly, the bacteriolytic activity in this hydrogel formulation persisted for an extended period. Kheljan et al. [185] developed a hydrogel containing a phage cocktail against *Pseudomonas aeruginosa* as an innovative dressing to support the treatment of infected wounds. The hydrogel used was composed of natural polymers: sodium alginate (4%) and carboxymethyl cellulose (2%), cross-linked with calcium chloride (CaCl_2_ at 5–10%) in both film and gel forms. In vitro and in vivo tests showed that phage hydrogels had antimicrobial efficacy comparable to antibiotics, while better supporting the wound healing process. Noteworthy is that the best results were obtained when phages were combined with ciprofloxacin, indicating their synergistic action and potential in the therapy of infected wounds. The improved wound healing observed in these studies further supports the biocompatibility of the applied carriers, as no irritation, necrosis, or delayed tissue regeneration was reported following topical application of phage-loaded formulations. Importantly, systemic exposure remains negligible due to localized application [175,184,185].

One of the major challenges in treating skin infections is ensuring phage penetration into deeper tissues. Campos et al. [186] utilized a sodium alginate-based hydrogel enriched with cholinium oleate, which acts as a skin penetration enhancer to immobilize a phage cocktail against *Acinetobacter baumannii*. Ex vivo experiments conducted on a pig skin model demonstrated that the addition of cholinium oleate significantly increased phage penetration into the tissue. In contrast to standard hydrogels, which showed no ability to penetrate the skin barrier, the incorporation of this compound led to an exponential increase in phage transport, making this technology a promising solution for treating hard-to-eliminate skin and subcutaneous tissue infections.

Creams represent another method for phage therapy delivery. Chang et al. [180] described a phage cream that exhibited significant antibacterial activity, although its phage release profile remained undefined. For topical applications, nonionic creams such as cetomacrogol water cream are recommended due to their superior compatibility with phages and improved biological stability compared to ionic bases. Chang et al. [180] presented a study in which phages PAC1–PAC10 were incorporated into a cream at a concentration of 2.5 × 10^8^ PFU/g to combat *Cutibacterium acnes*, a pathogen contributing to acne development. The preparation remained active for over 90 days at 4 °C, suggesting good shelf-life potential. While the use of wound dressings with immobilized phages is an intriguing concept, challenges related to formulation stability remain, and further research on storage conditions and optimization would be necessary before clinical application.

New biomaterials such as nanoemulsions and fibrin glues have also been employed for phage delivery in the treatment of infections [71]. Esteban et al. [187] demonstrated that phages in nanoemulsions were more stable and effective against *Staphylococcus aureus*, while Rubalskii et al. [188] confirmed that PA5 phages in fibrin glues maintained high titers for 11 days, effectively eliminating *Pseudomonas aeruginosa*. These findings suggest that biomaterials could serve as effective phage delivery systems for antibacterial therapies.

Films and polyelectrolyte microcapsules produced using layer-by-layer (LbL) technology (e.g., chitosan/alginate) represent another promising strategy. The choice of polymer parameters (pKa, charge density) and deposition conditions (ionic strength, pH) strongly influences encapsulation stability and release kinetics. At the site of infection, environmental changes such as pH shifts, variations in ionic strength, and enzymatic degradation of the polymer layers lead to gradual disassembly of the multilayer structure, enabling controlled phage release directly within biofilm-associated infection sites [189,190,191]. Recent studies have demonstrated effective control of *Salmonella enterica* using phages embedded in thin chitosan/alginate layers, with tunable release achieved by adjusting the number of LbL cycles and environmental ionicity. Although *Salmonella enterica* is not a skin pathogen, it was likely chosen due to its relevance in foodborne infections and as a model for enteric bacteria. The results are promising, showing significant reductions in bacterial counts and suggesting that LbL microcapsules could provide a controlled and efficient delivery system for phage-based antimicrobials [192,193,194].

In the future, combining bacteriophages with inorganic nanocarriers may attract increasing attention. Metal nanoparticles can act as carriers for bacteriophages, creating a hybrid system with enhanced antibacterial effect. Metal nanoparticles offer a large specific surface area, the ability to control ligand density, and facilitate the design of multivalent interactions that increase affinity for bacteria or biofilm matrices [195,196,197]. A specific therapeutic example is the use of phage conjugates and gold nanorods (AuNR) targeting *Pseudomonas aeruginosa*, which were applied topically to infected wounds in mice and activated with near-infrared radiation to rapidly reduce the number of bacteria. Importantly, chimeric phage-AuNR constructs were also designed to bind *Pseudomonas aeruginosa* among other Gram-negative pathogens [198]. In addition, silver (Ag) is known for its antibacterial properties and may act synergistically with phages. It was found that Ag nanoparticle-binding peptide (AgNP) on the capsid makes this system more effective at fighting pathogens than the phage alone. The synergistic activity of silver nanoparticles and bacteriophages led to the rapid dispersion of biofilm formed by *Staphylococcus aureus* [199]. Modified T7 phages armed with AgNP improved the elimination of *Escherichia coli* biofilm in vitro, which is more directly relevant for local applications against biofilms, e.g., on wound surfaces, than for systemic administration [200]. Manganese dioxide (MnO_2)_ nanostructures showed the greatest potential as phage carriers. These are responsive, catalytically active redox platforms that can be combined with phages in therapeutic nanocomposites. An excellent example is the phage-e6-MnO_2_ (PCM) nanocomposite, in which the phage provides host targeting and facilitates the delivery of the photosensitizer deep into the biofilm, while the MnO_2_ composite supports complex strategies such as photothermal therapy (PTT) and chemodynamic therapy (CDT). This leads to effective biofilm removal and, consequently, improved wound healing [201]. PCM systems have been evaluated in vivo in the context of wound healing associated with infection and discussed in the context of *Staphylococcus aureus* infection, which again fits best into a topical administration regimen combined with local irradiation [202].

While skin and wound infections remain the most common focus of topical phage therapy, similar challenges exist in ophthalmology. The eye presents a particularly complex environment due to constant tear fluid turnover, blinking, and the presence of protective barriers, all of which limit drug retention and bioavailability. Conventional eye drops are restricted by short residence time and limited penetration, whereas phage-based eye drops face the additional challenge of phage instability under unfavorable environmental conditions. To overcome these barriers, advanced nanotechnology-based carriers have been developed to enhance phage stability, prolong ocular residence, and enable controlled release [203].

In ocular applications, polymeric nanoparticles (PNPs), liposomes, nanoemulsions, dendrimers, nanofibers, and in situ gelling systems have been investigated for their ability to protect phages and improve delivery. PNPs, such as PLA-, PLGA-, or chitosan-based systems, encapsulate phages in nanospheres or nanocapsules, protecting them from degradation and improving mucoadhesion within the tear film [204]. Studies by Costa et al. [205] and Michalak et al. [206] confirmed that chitosan-coated alginate nanoparticles preserved phage viability and enhanced transcorneal permeability. Similarly, liposomes-owing to their phospholipid bilayer structure-shield phages from acidic pH and enzymatic degradation while mimicking biological membranes [177]. Colom et al. [96] and Singla et al. [68,112] demonstrated that liposome-encapsulated phages retained greater stability and prolonged antimicrobial activity against *Klebsiella pneumoniae* and *Staphylococcus aureus.*

Other nanocarriers are under experimental exploration [204]. Dendrimers, hyperbranched polymers such as PAMAM (poly(amidoamine) dendrimers), allow for multivalent loading of phage particles and functionalization, offering a platform for phage cocktails with reduced risk of resistance development [207]. Nanoemulsions, either oil-in-water or water-in-oil systems stabilized by surfactants, ensure high stability and have shown improved phage-bacteria interactions [187,208]. Nanofibers produced by electrospinning enable phage encapsulation in biodegradable matrices, acting as bioactive membranes with prolonged release, as demonstrated for *Pseudomonas* and *Mycobacterium* phages [209,210]. Finally, in situ gelling systems, which undergo sol-to-gel transition in response to ocular stimuli such as temperature or pH, prolong phage residence and reduce dosing frequency. For instance, Rahimzadeh et al. [211] developed thermosensitive gels carrying *Pseudomonas aeruginosa* phages, achieving superior efficacy in keratitis and conjunctivitis compared to standard eye drops. Taken together, these findings demonstrate that topical phage administration, whether for skin, wound, or ocular infections, benefits greatly from the integration of nanotechnology. By enhancing phage stability, prolonging release, and improving tissue penetration, nanocarrier-based systems significantly expand the therapeutic potential of phage therapy and address key challenges in localized infections caused by multidrug-resistant pathogens.

**Table 5:** The examples of phage therapy using topical administration.

Formulation	Phage	Infection	Model	Treatment Mode	Outcome	References
Gel	Phage FD3 in 2,5% Carbopol gel	*Cutibacterium acnes*	Mice model of *Cutibacterium acnes* induced lesions	Daily topical application of phage-loaded gel to the lesion site	Bacterial load reduction of 1.87 log_10_; lesion diameter, elevation and eschar scores significantly improved; no adverse events	[212]
	Phage cocktail (MR-5, MR-10), encapsulated in transferosomes	Methicillin-resistant *Staphylococcus aureus* (MRSA) SSTI	Rat soft-tissue infection model	Topical application in multiple doses	Faster MRSA clearance; higher phage titers maintained throughout treatment; infection resolved, enhanced phage stability and therapeutic efficacy compared to free phage	[93]
	Phage ZCKP8 gel (1:1 *v*/*v* with KY lubricating gel)	Multi-drug resistant *Klebsiella pneumoniae*	Rat full-thickness excisional wound model	Topical application of phage gel after 2 h infection period	Faster healing, histologically improved regenerated skin, and markedly reduced bacterial counts	[213]
Gauze dressing	Phage Phi_1	*Vibrio cholerae*	Infant rabbit cholera model	~10^9^ PFU Phi_1 given orally 6 h after infection	Reduction in bacterial counts in the intestines up to 4 log_10_ CFU/g. Reduced clinical signs of the disease	[101]
Liquid + cream	*Staphylococcus* bacteriophage and Pyobacteriophage cocktail	*Staphylococcus aureus*	Human case report of chronic skin infection	Gauze soaked with liquid phage applied to affected skin areas, followed by topical application of phage cream; 20 days of treatments for up to 6 months	Marked clinical improvement, reduced infiltration, erythema, and improved limb mobility; no allergic or adverse reactions	[214]
Hydrogel	Phage SAM-E.f incorporated into hydrogen (alginate, carboxymethyl cellulose and hyaluronic acid)	*Enterococcus faecalis*	Mouse model of wound infection	Topical application	Suppressed bacterial growth; improved wound closure and phage stability over time	[215]
	Phage 812K1/420 loaded on Gum Karaya-based hydrogel	Methicillin-resistant *Staphylococcus aureus*	Porcine deep wound infection model	Treatment applied to maintain a moist wound environment and support re-epithelialization; evaluated over 8 days	Significant bacterial load reduction (~2.5 log CFU/g) within one week; decrease in local inflammation; clear re-epithelialization and wound contraction; no adverse effects	[216]
	Phage cocktail loaded on carboxylmethylcellulose and alginate-based hydrogel	*Pseudomonas aeruginosa*	Mice model of wound infection	Topical application of phage-loaded hydrogel, daily or single dose	Rapid bacterial clearance; complete skin regeneration; daily treatment is more effective than single; improved survival and histology	[217]

## 5. Conclusions and Future Perspectives

**Figure 3:**
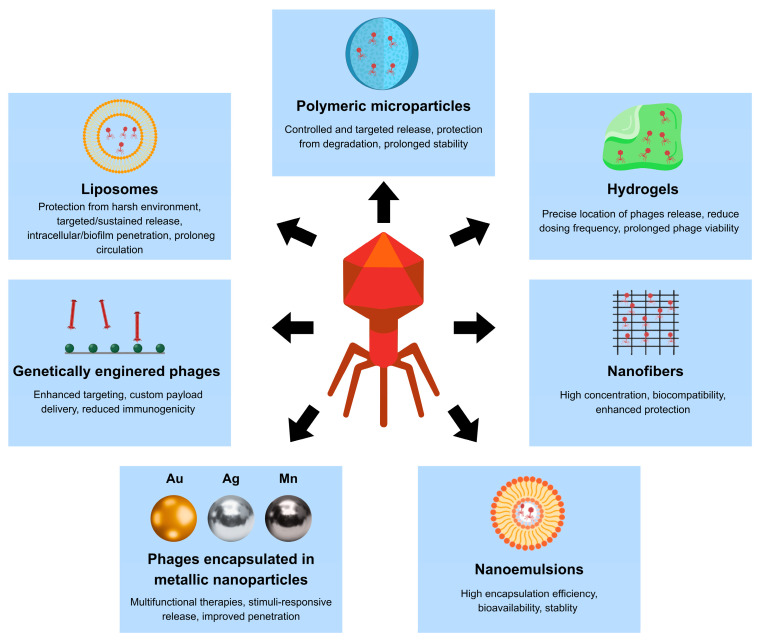
Key benefits of the most common phage delivery strategies.

The evolution of bacteriophage delivery strategy clearly shows a gradual development of a more functional design for specific clinical applications. A good phage formulation for therapy must overcome the problems arising from the nature of phages and posed by the environment (Figure 3).

In oral delivery, the field has moved beyond simple liquid suspensions toward protective strategies that enhance phage survival in the gastrointestinal tract, including pH-responsive encapsulation systems, polymer-based microcarriers, and lipid-derived formulations [67,77]. In systemic phage delivery, contemporary research increasingly emphasizes strategies to prolong circulation time, reduce rapid immune clearance, and improve tissue penetration [68]. Approaches such as liposomal encapsulation, polymeric nanoparticles, PEGylation, and other carrier-assisted delivery systems are being explored to modulate phage pharmacokinetics and biodistribution [218,219].

The development of hydrogel-based platforms opens new perspectives in the treatment of skin infections, with benefits including local delivery, increased retention at the wound site and protection of phage activity. Hydrogels maintain hydration balance in the wound, which is also important for the stabilization of phages, making these solutions especially promising for future regenerative medicine. The integration of nanotechnology and phage therapy may improve treatment efficacy via promoting deeper biofilm penetration in biofilm-associated wounds [128].

For bone and joint infections, the field is converging on highly personalized, predominantly local delivery using biomaterial carriers combined with surgery and antibiotics [63,127]. Novel phage-based materials support sustained contact between phages and bacteria adhered to the bone, which favours self-replication at the infection site. Pharmaceutical formulations, such as three-dimensional networks of polymers, bone cement, implant coating or nanoparticles, have improved the therapeutic potential for complex osteoarticular infections. Current challenges in 3D-printing techniques for bone tissue engineering open new research directions in the treatment of infected bones and the regeneration of bone tissue. 3D-printed materials can be specifically designed by producing patient-oriented scaffolds. However, in vivo and clinical trials are essential for the translation of laboratory findings into practical applications.

Phage therapy against pulmonary infections is particularly focused on nebulized liquids and dry powder inhalers because they are easy to produce and capable of delivering a high concentration into the respiratory environment. Currently, liposome-based formulations have emerged as a promising alternative for pulmonary administration [220]. Encapsulation in liposomes offers protection from external stressors during manufacturing and reduces phage cellular uptake in lung epithelial cells, which results in more viable phages for killing extracellular bacteria, e.g., *Pseudomonas aeruginosa* [114]. However, the diversity of study design, dosing regimen and timing of administration makes comparative analysis difficult. Key pharmacokinetic parameters like phage deposition in lungs, interactions with pulmonary defence mechanisms remains poorly characterized in human cases.

It is worth noting that phage delivery strategies are not equivalent in terms of manufacturing complexity, scalability or economic feasibility [67]. Advanced delivery platforms can substantially increase production complexity and cost, while posing challenges for large-scale manufacturing and quality control [221,222]. Additionally, the biological diversity of bacteriophages may prove to be a key factor determining unequal susceptibility to processing, formulation and storage conditions [67].

Storage stability and logistics are further pivotal factors determining the transition of phage preparations from the verification stage to clinical use. Typically, liposomes and hydrogels are distributed under refrigerated and light-protected conditions to minimize titer decline [176]. Freeze-drying or spray-drying processes with appropriate stabilizers may simplify the distribution and storage of phage formulations by reducing reliance on the cold chain [67,77,87]. Simultaneously, the diversity of bacteriophages results in formula-specific sensitivity to processing and storage-induced factors [67]. It is imperative that stability is verified on an individual basis using appropriate tests.

Overall, phage delivery systems hold immense promise in advanced therapeutic strategies. However, to overcome current limitations, continued research is needed on standardized methodologies and pharmacokinetics to fully exploit the potential of bacteriophage-based antimicrobials across diverse applications.

## Figures and Tables

**Table 1:** The examples of phage therapy administered via the oral route.

Formulation	Phage	Infection	Model	Treatment Mode	Outcome	References
Liposomes	Cocktail of phages (UAB_Phi20, UAB_Phi78 and UAB_Phi87) in cationic liposomes	*Salmonella* *typhimurium*	Broiler chicken model	100 µL of liposome-encapsulated cocktail (10^10^ PFU/mL) for 8 days by oral gavage	Prolonged intestinal residence time of the encapsulated phages and protection against *Salmonella* for at least 1 week. No signs of intestinal irritation or systemic toxicity during the treatment period	[96]
Hydrogels	Cocktail of phages (EFDG1 and EFLK1) formulated with poloxamer P407	*Enterococcus faecalis*	Rat root canal infection model	Single topical application during endodontic treatment (from ~10^9^ PFU/mL initially to ~10^3^ PFU/mL after 28 days)	The reduction (95–99%) of viable *Enterococcus faecalis* and biofilm mass in the canal after 3 weeks	[97]
Polymer-phage formulation	Phage A221 encapsulated in alginate	*Escherichia coli*	Weaned piglets	5 mL of encapsulated phage (10^9^ PFU/mL) for 7 days	Reduced bacterial load in tissues and intestinal lesions. Increased body weight of animals	[98]
	Phage SP4 encapsulated in xanthan gum	*Salmonella enteritidis*	Salmonellosis chicks model	0.5 g of microcapsules (3 × 10^10^ PFU/g) in feed	Reduced *Salmonella* colonization in the intestines. Improved therapeutic effect compared to free phages	[99]
	Phage SMHBZ8 in hydroxypropyl cellulose	*Streptococcus mutans*	Murine caries model	SMHBZ8 phage suspension (~10^8^ PFU/mL) by oral swab every 48 h for 42 days	Prevented dental carious lesion formation	[100]
Liquid phage suspension	Phage Phi_1	*Vibrio cholerae*	Infant rabbit cholera model	~10^9^ PFU Phi_1 given orally 6 h after infection	Reduction in bacterial counts in the intestines up to 4-log_10_ CFU/g. Reduced clinical signs of the disease	[101]
	Cocktail of phages (phiCDHM1, phiCDHM2, phiCDHM5 and phiCDHM6)	*Clostridium difficile*	Hamster model of acute infection	0.8 mL of single phage or phage cocktail (10^8^ PFU/mL) every 8 h until 36 h	The phage cocktail prolonged the time to onset of severe disease by ~33 h	[102]
Commercial capsules	*Escherichia coli*-targeting phage cocktail (LH01, LL5, T4D and LL12) in PreforPro^®®^ capsules	Overall gut microbiota	Human randomized clinical trial, gastrointestinal issues	One 15 mg capsule (10^6^ PFU/mL) per day for 28 days	No adverse events attributed to phages. Selective reduction in target pathogens without disruption of gut microbiota	[103]

## Data Availability

Data sharing is not applicable. No new data were created or analyzed in this study.
